# Digital Technologies in Cardiac Rehabilitation for High-Risk Cardiovascular Patients: A Narrative Review of Mobile Health, Virtual Reality, Exergaming and Virtual Education

**DOI:** 10.3390/jcm15031193

**Published:** 2026-02-03

**Authors:** Aleksandra Rechcińska, Barbara Bralewska, Marcin Mordaka, Tomasz Rechciński

**Affiliations:** 1 Independent Researcher, 90-014 Lodz, Poland; 2 SP ZOZ Central Clinical Hospital, Medical University of Lodz, 92-213 Lodz, Poland; 3 Holy Family Hospital Medical Center, 90-302 Lodz, Poland; 4 1st Department of Cardiology, Medical University of Lodz, 91-347 Lodz, Poland

**Keywords:** cardiac rehabilitation, digital technologies, mHealth

## Abstract

**Background:** Cardiac rehabilitation (CR) is a key component of secondary prevention after acute coronary events, coronary and valve interventions, and device implantation, yet participation and long-term adherence remain suboptimal. Digital technologies offer the potential to extend CR beyond the centre-based model and to support more flexible, patient-centred care. **Methods:** This narrative “review on a systematic backbone” synthesizes original clinical studies published between 2005 and 2025 that evaluated the use of digital technologies as an integral part of CR in adults after myocardial infarction, revascularization, valve procedures or implantation of cardiac devices. Interventions were grouped into four categories: mobile health (mHealth) and tele-rehabilitation, virtual reality (VR) and exergaming, virtual education platforms, and other multi-component digital CR solutions. Only original studies with clinical, functional, or patient-reported outcomes were included. **Results:** Twenty-one studies on the categories mentioned above met the eligibility criteria. mHealth-enabled home-based or hybrid CR programs consistently achieved improvements in functional capacity and physical activity that were broadly comparable to centre-based CR, with generally high adherence. VR and exergaming interventions were feasible and safe, produced at least similar functional gains, and showed more consistent benefits as far as anxiety levels and engagement levels. Virtual education platforms delivered knowledge and produced behaviour change similar to traditional education and, in some studies, supported better control of blood pressure and lipids. Comprehensive digital CR platforms improved risk-factor profiles and quality of life to a degree comparable with face-to-face CR. **Conclusions:** Digital technologies can credibly support core objectives of CR in high-risk patients and expand access, but must be implemented as a complement to, rather than a replacement for, multidisciplinary, patient-centred rehabilitation.

## 1. Introduction

Cardiac rehabilitation (CR) is a cornerstone of secondary prevention after acute coronary syndromes, coronary revascularization procedures, valve interventions, and implantation of cardiac devices, yet participation and long-term adherence remain suboptimal worldwide. Traditional centre-based CR programs face persistent barriers, including limited geographical access, transportation issues, time constraints related to work and family responsibilities, and limitations in reimbursement in some health systems. These barriers particularly affect older adults, patients with multiple comorbidities, and individuals living in rural or socioeconomically deprived areas.

In parallel, the last two decades have seen a rapid expansion of digital health technologies across cardiovascular medicine, including mobile health tools, remote monitoring, virtual reality, exergaming, and web-based educational platforms. Digital technologies offer new opportunities to redesign CR delivery by extending care beyond the hospital or outpatient centre, enabling continuous monitoring, and supporting behavioural change in real-life environments. Scientific statements and expert position papers have highlighted technology-enabled CR as a key strategy to increase reach, personalization, and efficiency of secondary prevention.

Within this field, several distinct but overlapping modalities can be identified. Mobile health (mHealth) and tele-rehabilitation interventions use smartphones, wearable sensors, and web platforms to deliver exercise prescriptions, education, and remote coaching, often as part of hybrid CR models that combine in-person and home-based components [1,2]. Virtual reality (VR) and exergaming systems create immersive or game-like environments that may enhance motivation, reduce anxiety, and provide real-time feedback during exercise sessions. Virtual education platforms and online group programs can complement or replace traditional face-to-face educational sessions, potentially improving flexibility and scalability [3].

Despite rapid development, the evidence base for digital technologies in CR remains heterogeneous, with substantial variation in target populations, technological components, intervention intensity, and reported outcomes. Existing reviews often focus on broader digital health in cardiovascular disease or on a single modality, with less emphasis on high-risk post-event or post-procedure patients such as those after valve interventions or device implantation. In addition, many reports mix full-scale clinical interventions with studies of early-stage prototypes and of feasibility, which complicates translation into routine practice [4,5].

The aim of this review was to synthesize original clinical studies published between 2005 and 2025 that evaluated digital technologies supporting CR in adult patients after acute coronary events, coronary or valve interventions, or implantation of cardiac devices. The review focuses on four categories of digital modalities: (1) mobile health (mHealth) and tele-rehabilitation, (2) VR and exergaming systems, (3) virtual education platforms, and (4) other digital technologies directly supporting CR delivery. Using methods aligned with contemporary reporting standards for systematic reviews, the review describes the characteristics of these interventions, summarizes their effects on functional, clinical, and patient-reported outcomes, and identifies key gaps and priorities for future research and implementation.

## 2. Materials and Methods

### 2.1. Eligibility Criteria

The review included quantitative clinical studies evaluating digital technologies used to support CR in adults. Populations of interest were adults (≥18 years) with established cardiovascular disease participating in CR after acute coronary events, coronary revascularization, valve interventions, or implantation of cardiac devices such as pacemakers, implantable cardioverter-defibrillators, or cardiac resynchronization systems.

Eligible interventions were CR programs or components of CR that incorporated digital technologies as an integral element, classified into four categories:

1. mHealth or tele-rehabilitation (for example, smartphone applications, wearable activity monitors, or web platforms used for exercise prescription, monitoring, or coaching);
2. VR or exergaming systems applied during exercise or rehabilitation sessions;
3. virtual education platforms (for example, web-based curricula, online group education, or virtual-world educational programs);
4. other digital technologies directly supporting CR delivery (for example, comprehensive digital CR platforms, or prototypes of new devices and software tested in CR patients).

Comparators could include conventional centre-based CR, hybrid CR without a digital component, other forms of usual care, or no structured CR. The review considered randomized controlled trials, quasi-experimental studies, cohort studies with a comparator group, and feasibility or pilot studies of new devices or applications, provided that they reported at least one clinical, functional, or patient-reported outcome.

We conducted a structured search in PubMed/MEDLINE using a combination of controlled vocabulary and free-text terms related to cardiac rehabilitation, telemedicine, mobile health, remote monitoring and virtual reality, combined with Boolean operators and filters for adult human studies.

The review excluded narrative and systematic reviews, meta-analyses, case reports, editorials, letters, study protocols without clinical outcome data, conference abstracts without full papers, and purely technical reports with no patient involvement. Only articles published in English with an available abstract were included. The time frame was from 1 January 2005 to 5 December 2025.

This review was conducted using systematic search, selection and data extraction procedures aligned with the PRISMA 2020 framework, although we did not prospectively register a protocol [6].

### 2.2. Information Sources and Search

The primary search was performed in MEDLINE (PubMed), combining terms related to CR (“cardiac rehabilitation”) with terms related to digital technologies (“digital technology”, “mobile health”, “VR”, “exergaming”, “virtual education”, restricted to the predefined time frame and English-language publications, and excluding reviews and case reports.

### 2.3. Study Selection and Data Extraction

All records identified through the search were imported into a reference management system and duplicates were removed. Titles and abstracts were screened against the eligibility criteria, followed by full-text assessment of potentially relevant articles. Reasons for exclusion at the full-text stage were documented. 

For each included study, the following data were extracted using a standardized form: study design, country and setting, sample size and patient characteristics (including index event or index procedure), CR setting and phase, category of digital modality, details of the intervention and comparator, follow-up duration, and reported outcomes. Outcomes of interest included functional capacity (for example, 6 min walk distance—6MWD, peak oxygen uptake), clinical events (for example rehospitalizations, mortality), cardiovascular risk factors, quality of life, psychological measures, adherence and completion, and safety events.

Given the expected heterogeneity in populations, technologies, and outcomes, no formal meta-analysis was planned. Instead, results are presented in a structured narrative synthesis.

## 3. Results

Out of 1075 articles, a total of 21 original studies met the inclusion criteria and were included in the narrative synthesis. Based on their primary technological component, these studies were grouped into four categories: mHealth and tele-rehabilitation interventions, VR and exergaming systems, virtual education platforms, and other digital technologies directly supporting CR delivery. Most studies enrolled patients after acute coronary syndromes or coronary revascularization, with a smaller number including individuals after valve interventions, heart failure decompensation, or device implantation.

### 3.1. Mobile Health and Tele-Rehabilitation Interventions

Across the included studies, mHealth and tele-rehabilitation interventions most commonly combined smartphone applications, wearable activity monitors, and web-based platforms to deliver or augment phase II of home-based or hybrid CR [7,8,9]. Typical components included remote exercise prescriptions, app-based feedback on physical activity, teleconsultations with CR staff, and digital monitoring of vital signs and of cardiovascular risk factors.

Randomized and controlled studies in post-myocardial infarction and post-revascularization populations consistently showed clinically relevant gains in functional capacity with mHealth-enabled CR. In representative trials, mean 6MWD increased more in mHealth-supported groups than in controls receiving centre-based CR or usual care, and similarly peak oxygen uptake improved by around 2–3 mL/kg/min in intervention groups compared with smaller gains in comparators [10,11]. These functional benefits were accompanied by increases in daily step counts or weekly minutes of moderate-to-vigorous physical activity, as well as by stable or improved blood pressure and lipid profiles [12].

Adherence and completion rates in mHealth-enabled CR were generally high, frequently in the range of 75–80% or more, suggesting that remote monitoring, app-based reminders, and flexible scheduling can mitigate traditional access barriers such as travel distance and time constraints. In heart failure populations, tele-rehabilitation and digital monitoring approaches were feasible and acceptable, with signals toward improved functional capacity and symptom burden, although data on long-term rehospitalizations and mortality remain limited. Overall, mHealth-supported home-based or hybrid CR turn out to be capable of delivering functional and behavioural outcomes at least comparable to those of conventional centre-based CR, while offering added flexibility and accessibility.

Detailed study characteristics and outcomes are provided in Supplementary Table S1.

### 3.2. Virtual Reality and Exergaming Systems

Original studies on VR and exergaming in CR evaluated both non-immersive, screen-based systems and immersive head-mounted devices used to support aerobic or cycling exercise sessions or relaxation modules. Participants were mainly patients with coronary artery disease in phase II of outpatient CR, with some studies involving heart failure patients during early or maintenance rehabilitation [13,14].

Several VR-augmented programs demonstrated substantial improvements in 6MWD in both VR/exergaming and control groups, with VR arms often showing numerically larger gains, although between-group differences were not always statistically significant. These findings indicate that VR-supported exercise can achieve functional benefits similar to standard CR, while offering a different experiential format [15,16,17].

Psychological outcomes were a prominent focus in multiple studies. VR-based relaxation or game-like scenarios integrated into CR led to greater reductions in anxiety and emotional tension compared with standard CR alone, even when functional gains were comparable. Immersive VR interventions in particular showed larger decreases in anxiety scores on validated scales at program completion. Early exergaming prototypes that combined VR with real-time biometric monitoring and semi-automated session management were feasible and well tolerated, with high usability ratings and no major adverse events, suggesting that these systems can be safely integrated into CR workflows [18].

Detailed study characteristics and outcomes are provided in Supplementary Table S2.

### 3.3. Virtual Education Platforms

Studies on virtual education in CR evaluated web-based curricula and online group programs, usually delivered alongside standard exercise training during phase II or III of CR. Interventions included synchronous group videoconferencing, structured online learning platforms, and, in some cases, VR-enhanced educational content applied during exercise [19].

Randomized comparisons of virtual versus in-person group education showed that online programs achieved similar improvements in disease-specific knowledge and self-reported health behaviours, including medication adherence and dietary changes. Short-term functional outcomes, such as 6 min walk distance, were generally similar between virtual and conventional education groups, consistent with the idea that the main added value of virtual formats lies in flexibility and accessibility rather than in superior exercise effects.

In programs where virtual or blended education constituted a central component, improvements in risk-factor control paralleled gains in knowledge and self-management. Some studies reported a high proportion of participants achieving target control of blood pressure and LDL cholesterol after virtual or blended education, sometimes numerically higher than in groups receiving conventional education alone. Technology-supported home-based CR interventions that incorporated structured remote education, alongside exercise and monitoring, demonstrated modest reductions in systolic blood pressure and favourable changes in lipid parameters compared with usual care, confirming that digital education plays an important role for in cardiometabolic risk-factor management [20].

Qualitative and mixed-methods analyses highlighted improved accessibility, a sense of empowerment, and the convenience of participating from home as major facilitators of virtual education. In contrast, usability issues, technical problems, and variable social support were recurring barriers that could affect engagement and sustained participation. Overall, virtual education platforms appear capable of delivering knowledge and behaviour change outcomes similar to those of traditional education, with greater flexibility but also with new technology-related challenges.

Detailed study characteristics and outcomes are provided in Supplementary Table S3.

### 3.4. Other Digital Technologies Supporting Cardiac Rehabilitation

A smaller number of original studies evaluated comprehensive digital CR platforms that combined multiple functionalities, such as remote exercise prescription and monitoring, interactive educational modules, risk-factor tracking, and secure communication channels with the CR team. These systems were usually deployed as home-based or hybrid programs intended to provide a fully digital pathway paralleling centre-based CR.

Large cohort studies of such digitally enabled CR programs reported significant improvements in cardiovascular risk factors, including blood pressure, lipid profile, and body weight, with effect sizes comparable to or slightly better than those observed in conventional face-to-face CR. Rehospitalization rates, hospital bed-days, and mortality at one year were generally similar between digital and centre-based CR, suggesting equivalent impact on hard outcomes within the available follow-up [21].

Longer-term observational data indicated that structured digital CR pathways may support sustained lifestyle changes and preservation of health-related quality of life, while enhancing patient empowerment and self-management through continuous access to personalized information and feedback. At the same time, these complex platforms highlight the importance of health-system integration, including interoperability, with electronic health records, data protection, and appropriate reimbursement, to enable broader implementation [22,23,24].

Detailed study characteristics and outcomes are provided in Supplementary Table S3.

## 4. Discussion

This review suggests that digital technologies can reliably support the core objectives of CR in high-risk cardiovascular patients while addressing several long-standing barriers associated with centre-based programs. Across different modalities, the majority of digital interventions delivered functional, behavioural, and risk-factor outcomes that were broadly similar to those achieved with conventional CR. As a result, the key question is increasingly shifting from whether digital CR works to how these tools can be implemented safely, equitably, and sustainably in routine practice.

A major strength of digital solutions is their ability to extend rehabilitation into patients’ everyday environments. mHealth-enabled home-based and hybrid programs facilitate flexible scheduling, continuous feedback, and closer alignment with real-world physical activity patterns. VR and exergaming appear particularly useful for addressing psychological domains such as anxiety and engagement, which are often under-recognized, yet clinically important components of CR. Virtual education and blended telerehabilitation programs can reinforce guideline-recommended risk-factor control and support long-term behaviour change at scale.

However, the existing evidence base has important limitations. Many studies are single-centre, include modest sample sizes, and employ heterogeneous intervention designs and outcome measures, which limits comparability and the strength of inferences that can be drawn. Long-term data on hard endpoints such as rehospitalizations, mortality, and sustained adherence beyond one to two years remain scarce. In addition, digital literacy, access to suitable devices, reliable internet connectivity, and variability in social and family support represent critical determinants of who can benefit from digital CR.

Digital health literacy is an increasingly recognized determinant of who can realistically participate in, and benefit from, technology-enabled cardiac rehabilitation, and it has direct implications for selection bias, effect estimates, and the external validity of digital intervention trials. In contemporary cardiovascular cohorts, the overall level of eHealth literacy is often modest, and large survey studies consistently show that older age, lower educational attainment, lower income and rural residence are associated with substantially poorer skills in finding, understanding and applying online health information. When digital interventions require patients to own a smartphone or computer, have reliable internet access, navigate apps or web platforms and interpret digital feedback, these structural and cognitive prerequisites act as an additional set of inclusion criteria on top of the usual clinical eligibility, even when they are not explicitly labelled as such. As summarized by Wells and others, this means that trials of apps, telemonitoring programs, patient portals and e-learning platforms in cardiovascular disease tend to preferentially recruit younger, better educated and more health-activated participants, while systematically under-representing precisely those groups that bear the highest burden of cardiovascular morbidity and mortality, such as older adults, people with low socioeconomic status, limited formal education or limited language proficiency [25,26].

This pattern introduces several, partly overlapping, sources of bias. First, there is a clear selection bias or “healthy user” effect: patients with higher digital health literacy are more likely to consent to take part in a trial, to complete onboarding, to remain engaged with the technology, and to adhere to the prescribed rehabilitation program. These same individuals also tend to have more favourable baseline behaviours (healthier diet, greater physical activity, better medication adherence) and stronger self-management skills, which are independently associated with improved clinical outcomes irrespective of the digital tool itself. If a digital intervention trial enrols mainly these digitally and behaviourally advantaged participants, the observed improvements in secondary outcomes such as CR attendance, exercise minutes, risk factor control or quality of life may partly reflect the underlying characteristics of the study population rather than the intrinsic efficacy of the technology. Second, differential attrition can reinforce this bias: participants with lower eHealth literacy are more likely to drop out because of frustration with the technology, technical failures or lack of perceived benefit, whereas those with higher literacy persist and contribute a disproportionate share of the outcome data. Analyses that are restricted to completers, or that inadequately address missing data, therefore risk further overestimating the effect size in a way that is rarely visible from the trial report alone [27].

A related concern is limited external validity. Even when randomization within a trial is robust and internal validity is high, the results may not generalize to the broader population of patients eligible for cardiac rehabilitation if a sizeable fraction of real-world patients would be unable or unwilling to use the tested technology. Wells highlights that many digital cardiovascular studies either do not measure eHealth literacy at all, or use only crude proxies such as device ownership or self-reported internet use; as a result, there is often no way to quantify how far the trial sample is skewed towards digitally competent users. In addition, very few trials prespecify subgroup analyses by digital literacy, so it remains largely unknown whether intervention effects are comparable, attenuated or absent in patients with low literacy. From a health equity perspective, this is important: if the benefits of digital cardiac rehabilitation accrue predominantly to patients who already have relatively high levels of health literacy, digital interventions could inadvertently widen existing disparities in cardiovascular outcomes instead of narrowing them. Conversely, some observational work suggests that patients with lower baseline digital literacy may actually have the largest absolute room for improvement if the intervention is specifically tailored to their needs and accompanied by structured support, but this hypothesis has rarely been tested in high-quality trials [28].

In light of this evidence, differential digital health literacy should be explicitly acknowledged as a major methodological limitation of the current evidence base on digital cardiac rehabilitation, rather than as a minor or incidental issue. At a minimum, future studies should routinely assess eHealth literacy using validated instruments (for example eHEALS or comparable scales), report the distribution of scores in their samples, and consider digital literacy in both design and analysis (e.g., stratified randomisation, predefined subgroup analyses, and adjustment for literacy as a covariate in multivariable models). Just as importantly, digital interventions themselves must be designed with inclusivity in mind: interfaces should be simplified, cognitive load reduced, language adapted, and additional human support (training sessions, telephone coaching, in-person troubleshooting) built into the model of care, so that patients with limited experience of technology are not systematically excluded from participation. Wells also points to emerging interventions that first aim to improve patients’ eHealth literacy and digital self-efficacy and then layer disease-specific digital programs on top; meta-analytic data suggest that such approaches can meaningfully increase eHealth literacy compared with usual care, although the downstream impact on hard cardiovascular outcomes still needs to be established. Taken together, these insights imply that the positive results observed in many digital cardiac rehabilitation trials— including those synthesized in our review—are likely to represent best-case estimates in digitally capable, motivated patients, and should therefore be interpreted with caution when extrapolated to older, socioeconomically disadvantaged or technologically inexperienced populations who currently remain under-represented in the evidence base [29].

Another important limitation is that we did not perform a formal, tool-based risk-of-bias assessment (e.g., RoB 2 or ROBINS-I) for the individual studies, mainly because of the pronounced heterogeneity in designs, interventions and outcomes across the included literature. As a result, our synthesis cannot provide a graded comparison of the internal validity or certainty of evidence between different digital modalities, and the strength of individual findings should be interpreted with caution. Furthermore, most available studies are single-centre, involve relatively small samples and short follow-up, and focus predominantly on functional capacity, risk factor control and psychological outcomes, whereas data on hard clinical endpoints such as mortality, rehospitalizations and long-term adherence beyond one to two years are scarce or entirely lacking, reflecting the early stage of this field rather than a gap in our search. Taken together, these factors limit the possibility of conducting a meaningful quantitative meta-analysis and restrict the generalisability of our conclusions, underscoring the need for larger, methodologically robust trials with extended follow-up in more diverse cardiac populations.

These factors underline that technology cannot replace the human elements of CR: multidisciplinary expertise, therapeutic relationships, and individualized clinical judgment remain central.

From a system perspective, successful deployment of digital CR requires integration with electronic health records, clear clinical pathways, and appropriate reimbursement models. Without these structural supports, even well-designed digital interventions remain confined to pilot projects with limited impact on the population. Future research should prioritize larger, pragmatically designed studies with explicit attention to implementation, equity, and cost-effectiveness, and should deliberately include populations that have been under-represented so far, such as patients after valve interventions and device implantation.

Recent systematic work by Moreira et al. demonstrates that conventional, centre-based CR consistently improves the physical component of quality of life after coronary events, while effects on mental domains are more variable and often less pronounced. This pattern is broadly aligned with our observations in digital CR, where functional gains are clear but psychological improvements are heterogeneous and frequently underreported. In parallel, emerging mHealth studies in heart failure suggest that mobile tools can support self-care maintenance and patients’ perceived security over longer periods, yet the impact on global quality of life remains uncertain and may diminish with time. Together, these data underscore the need for future digital CR trials to use standardized PROMs longitudinally and to treat patient reported experience and quality of life as core outcomes rather than secondary add-ons [30,31].

We are aware also some methodological limitations of presented review.

Our search strategy was restricted to PubMed/MEDLINE, which offers broad coverage of cardiovascular journals and applies rigorous journal-selection and indexing standards through the National Library of Medicine, thereby providing a relatively well-curated and reproducible corpus of clinical research. However, we acknowledge that relying on a single database introduces potential database bias and may have led to the omission of relevant studies indexed exclusively in other sources (e.g., Embase, Web of Science, regional databases or grey literature), particularly smaller or region-specific digital health and telerehabilitation projects. Consequently, the body of evidence synthesized in this review should be interpreted as reflecting the subset of digital cardiac rehabilitation studies captured in PubMed/MEDLINE, and future reviews could strengthen completeness by incorporating additional databases and systematic grey-literature searches.

Our review, as a „narrative review on a systematic backbone”, combines systematic identification and selection of studies with a predominantly narrative synthesis; therefore, we chose to structure reporting according to PRISMA-type items rather than formally applying SANRA, which is primarily a quality-assessment tool for non-systematic narrative reviews [32].

## 5. Conclusions

Digital technologies have progressed from experimental adjuncts to credible delivery options for contemporary CR. mHealth-enabled home-based and hybrid programs, VR and exergaming systems, virtual education platforms, and comprehensive digital CR pathways together form a flexible toolkit that can support more patient-centred, accessible models of rehabilitation. In the studies reviewed, these approaches generally achieved clinical and behavioural outcomes comparable to those of centre-based CR, with added benefits regarding accessibility, engagement, and in some cases psychological well-being.

At the same time, digital CR must be understood as a complement to—not a substitute for—the human, multidisciplinary nature of rehabilitation. Clinicians, nurses, physiotherapists, psychologists, and other professionals remain essential to patients’ assessment, their risk stratification, shared decision-making, and ongoing support. Future work should move beyond proof-of-concept studies towards comprehensive, long-term evaluations embedded in routine care, with careful attention to patient selection, equity of access, data protection, and integration within health-system structures. Only the combination of modern technologies with an experienced team and careful care for a specific person will allow us to fully utilize the potential of digital cardiac rehabilitation.

## Data Availability

No new data were created or analyzed in this study.

## Supplementary Materials

The following supporting information can be downloaded at: https://www.mdpi.com/article/10.3390/jcm15031193/s1, Table S1. Mobile health and tele-rehabilitation interventions—detailed study characteristics and outcomes; Table S2. Virtual reality and exergaming systems—detailed study characteristics and outcomes; Table S3. Virtual education platforms and other digital technologies supporting cardiac rehabilitation—detailed study characteristics and outcomes.

## Abbreviations

The following abbreviations are used in this manuscript:

AMI	Acute myocardial infarction
CPET	Cardio-pulmonary exercise test
CR	Cardiac rehabilitation
DASI	Duke Activity Status Index
LDL	Low density lipoprotein
mHealth	Mobile health
PCI	Percutaneus coronary intervention
PROM	Patient-reported outcome measures
QoL	Quality of life
VR	Virtual reality
6MWD	6 min walk distance

## References

[1] Sassone B., Fuca’ G., Pedaci M., Lugli R., Bertagnin E., Virzi’ S., Bovina M., Pasanisi G., Mandini S., Myers J. (2024). Analysis of Demographic and Socioeconomic Factors Influencing Adherence to a Web-Based Intervention Among Patients After Acute Coronary Syndrome: Prospective Observational Cohort Study. JMIR Cardio.

[2] Schuuring M.J., Treskes R.W., Castiello T., Jensen M.T., Casado-Arroyo R., Neubeck L., Lyon A.R., Keser N., Rucinski M., Marketou M. (2024). Digital solutions to optimize guideline-directed medical therapy prescription rates in patients with heart failure: A clinical consensus statement from the ESC Working Group on e-Cardiology, the Heart Failure Association of the European Society of Cardiology, the Association of Cardiovascular Nursing & Allied Professions of the European Society of Cardiology, the ESC Digital Health Committee, the ESC Council of Cardio-Oncology, and the ESC Patient Forum. Eur. Heart J. Digit. Health.

[3] Lunz L., Würth S., Kulnik S.T. (2025). Health Care Professionals’ Use of Digital Technology in the Secondary Prevention of Cardiovascular Disease in Austria: Online Survey Study. JMIR Cardio.

[4] Al-Dhahir I., Breeman L.D., Faber J.S., Wentzel J., van den Berg-Emons R.J.G., Kraal J.J., Janssen V.R., Kraaijenhagen R.A., Visch V.T., Chavannes N.H. (2025). Content evaluation of the inclusive eHealth guide: How to develop interventions for people with a lower socioeconomic position?. Front. Digit. Health.

[5] Hughes J.W., Berry R., Brown T.M., Carlin B., Drwal K., Keteyian S.J., Prince D.Z., Wu W.C. (2025). Consensus Statement on the Virtual and Remote Delivery of Cardiac and Pulmonary Rehabilitation and Their Components. J. Cardiopulm. Rehabil. Prev..

[6] Page M.J., McKenzie J.E., Bossuyt P.M., Boutron I., Hoffmann T.C., Mulrow C.D., Shamseer L., Tetzlaff J.M., Akl E.A., Brennan S.E. (2021). The PRISMA 2020 statement: An updated guideline for reporting systematic reviews. Rev Esp Cardiol (Engl. Ed.).

[7] Harpula J., Kalańska-Łukasik B., Głód G., Gąsierkiewicz P., Barnaś O., Danioł M., Godek P., Wita K., Kowalska M., Wojakowski W. (2025). Feasibility and efficacy of mobile app implementation among patients with acute myocardial infarction enrolled in coordinated cardiac rehabilitation program. Front. Digit. Health.

[8] Zeid S., Prochaska J.H., Schuch A., Tröbs S.O., Schulz A., Münzel T., Pies T., Dinh W., Michal M., Simon P. (2025). Personalized app-based coaching for improving physical activity in heart failure with preserved ejection fraction patients compared with standard care: Rationale and design of the MyoMobile Study. Eur. Heart J. Digit. Health.

[9] Harzand A., Alrohaibani A., Idris M.Y., Spence H., Parrish C.G., Rout P.K., Nazar R., Davis-Watts M.L., Wright P.P., Vakili A.A. (2023). Effects of a patient-centered digital health intervention in patients referred to cardiac rehabilitation: The Smart HEART clinical trial. BMC Cardiovasc. Disord..

[10] Hayn D., Sareban M., Höfer S., Wiesmüller F., Mayr K., Mürzl N., Porodko M., Puelacher C., Moser L.M., Philippi M. (2023). Effect of digital tools in outpatient cardiac rehabilitation including home training-results of the EPICURE study. Front. Digit. Health.

[11] Giggins O.M., Cullen-Smith S., Kenny E., Doyle J. (2024). Integrating the quantitative with the qualitative: Findings from a mixed methods cardiac rehabilitation exercise trial. Heart Rhythm O_2_.

[12] Wu Y., Yang F., Feng Y., Xu Q., Zhu H. (2024). Effectiveness of ‘Internet Plus’ remote management in improving cardiac rehabilitation outcomes in acute myocardial infarction patients. Am. J. Transl. Res..

[13] Blasco-Peris C., Alcolea Garrido J.P., Seguí B., Zaragoza R., Climent-Paya V., Fuertes-Kenneally L., Manresa-Rocamora A., Sanz-Rocher A., Baladzhaeva S., Sarabia J.M. (2025). Exergaming System for Exercise-Based Cardiac Rehabilitation in Patients with Heart Failure: Development and Usability Assessment Study of a Device Prototype. JMIR Serious Games.

[14] Jóźwik S., Cieślik B., Gajda R., Szczepańska-Gieracha J. (2021). The Use of Virtual Therapy in Cardiac Rehabilitation of Female Patients with Heart Disease. Medicina.

[15] Jóźwik S., Cieślik B., Gajda R., Szczepańska-Gieracha J. (2021). Evaluation of the Impact of Virtual Reality-Enhanced Cardiac Rehabilitation on Depressive and Anxiety Symptoms in Patients with Coronary Artery Disease: A Randomised Controlled Trial. J. Clin. Med..

[16] Gulick V., Graves D., Ames S., Krishnamani P.P. (2021). Effect of a Virtual Reality-Enhanced Exercise and Education Intervention on Patient Engagement and Learning in Cardiac Rehabilitation: Randomized Controlled Trial. J. Med. Internet Res..

[17] Szczepańska-Gieracha J., Jóźwik S., Cieślik B., Mazurek J., Gajda R. (2021). Immersive Virtual Reality Therapy as a Support for Cardiac Rehabilitation: A Pilot Randomized-Controlled Trial. Cyberpsychol. Behav. Soc. Netw..

[18] Sibilitz K.L., Tang L.H., Berg S.K., Thygesen L.C., Risom S.S., Rasmussen T.B., Schmid J.P., Borregaard B., Hassager C., Køber L. (2022). Long-term effects of cardiac rehabilitation after heart valve surgery—Results from the randomised CopenHeart_VR_ trial. Scand Cardiovasc. J..

[19] Brewer L.C., Kaihoi B., Schaepe K., Zarling K., Squires R.W., Thomas R.J., Kopecky S. (2017). Patient-perceived acceptability of a virtual world-based cardiac rehabilitation program. Digit. Health.

[20] Adachi T., Ashikawa H., Funaki K., Kondo R., Yamada S., Amano T. (2025). Feasibility of a Web-App-Based Remote Lifestyle Modification Program After Percutaneous Coronary Intervention. Int. Heart J..

[21] Lăcraru A.E., Busnatu Ș.S., Pană M.A., Olteanu G., Șerbănoiu L., Gand K., Schlieter H., Kyriazakos S., Ceban O., Andrei C.L. (2023). Assessing the Efficacy of a Virtual Assistant in the Remote Cardiac Rehabilitation of Heart Failure and Ischemic Heart Disease Patients: Case-Control Study of Romanian Adult Patients. Int. J. Environ. Res. Public Health.

[22] Indraratna P., Biswas U., McVeigh J., Mamo A., Magdy J., Vickers D., Watkins E., Ziegl A., Liu H., Cholerton N. (2022). A Smartphone-Based Model of Care to Support Patients with Cardiac Disease Transitioning from Hospital to the Community (TeleClinical Care): Pilot Randomized Controlled Trial. JMIR Mhealth Uhealth.

[23] Monturo C., Smith C., Bannon W.M., Brockway C. (2025). Community Cardiac Rehabilitation Program: Lessons Learned for Long-Term Outcomes. Worldviews Evid. Based Nurs..

[24] Oanesa R.D., Atluri N., Boyd A., Khoury S.R., Legeza V., Merritt J.G., McNair P., Mishra S.R., Ramsis M., Spaulding E.M. (2025). Human-Centered Design to Tailor Telehealth Cardiac Rehabilitation to Diverse Populations: The MCNAIR Study. J. Am. Heart Assoc..

[25] Wells R., Boulos L., Gray M., Keeping S.E., Devereaux E.J., Brauer-Chapin T., Hariharan A., Fera G., DeCoste K., Hickey M. (2026). Wide variability in studies reporting on digital education interventions for patients undergoing cardiac procedures: A patient-commissioned mixed methods systematic review. Heart Lung.

[26] Brørs G., Larsen M.H., Hølvold L.B., Wahl A.K. (2023). eHealth literacy among hospital health care providers: A systematic review. BMC Health Serv. Res..

[27] Barbati C., Maranesi E., Giammarchi C., Lenge M., Bonciani M., Barbi E., Vigezzi G.P., Dragoni M., Bailoni T., Odone A. (2025). Effectiveness of eHealth literacy interventions: A systematic review and meta-analysis of experimental studies. BMC Public Health.

[28] Maddula R., MacLeod J., McLeish T., Painter S., Steward A., Berman G., Hamid A., Abdelrahim M., Whittle J., Brown S.A. (2022). The role of digital health in the cardiovascular learning healthcare system. Front. Cardiovasc. Med..

[29] Höppchen I., Wurhofer D., Meschtscherjakov A., Smeddinck J.D., Kulnik S.T. (2024). Targeting behavioral factors with digital health and shared decision-making to promote cardiac rehabilitation-a narrative review. Front. Digit. Health.

[30] Moreira J., Bravo J., Aguiar P., Delgado B., Raimundo A., Boto P. (2024). Physical and Mental Components of Quality of Life after a Cardiac Rehabilitation Intervention: A Systematic Review and Meta-Analysis. J. Clin. Med..

[31] Miguel S.S.A., Frade A.I.A., Moreira J.M.A. (2024). The use of m-health to improve self-care in patients with heart failure. Eur. J. Cardiovasc. Nurs..

[32] Baethge C., Goldbeck-Wood S., Mertens S. (2019). SANRA-a scale for the quality assessment of narrative review articles. Res. Integr. Peer Rev..

