# Supplementary material for: Digital Technologies in Cardiac Rehabilitation for High-Risk Cardiovascular Patients: A Narrative Review of Mobile Health, Virtual Reality, Exergaming and Virtual Education
Source: J Clin Med. 2026 Feb 3;15(3):1193. doi: 10.3390/jcm15031193 (PMC12897756; doi:10.3390/jcm15031193)
Supplement: Supplementary file 1 [file jcm-15-01193-s001.zip › jcm-4101086-supplementary.pdf]

Table S1. Mobile health and tele-rehabilitation interventions - detailed study characteristics and outcomes

Author, year, (PMID)	Country	Population and index event	CR phase and setting	Digital intervention	Comparator	Follow-up duration	Main outcomes	Key quantitative results
Harpula et al., 2025 (PMID: 41281290)	Poland	103 survivors of AMI enrolled in a post-AMI comprehensive rehabilitation programme	Early outpatient phase following index hospitalization; home-based follow-up within a coordinated CR pathway	AHP-KOS mobile application supporting the coordination and conduct of outpatient CR visits (scheduling, reminders, documentation)	Standard coordinated care in the post-AMI programme without the mobile application	6 weeks after AMI	Completion of the first outpatient CR visit (adherence), duration of onsite visit; baseline cardiovascular risk profile	Use of the AHP-KOS app was associated with a higher completion rate of the first outpatient CR visit (91.7% vs 67.4%) and a shorter onsite visit duration (mean 8 vs 11 minutes) compared with standard coordinated care alone.
Zaid et al., 2025 (PMID: 40110212)	Germany	185 patients with heart failure with preserved ejection fraction (HFpEF) considered for a home-based physical activity intervention	Long-term management phase; home-based setting with study visits in a specialized centre	MyoMobile smartphone app with personalized physical activity coaching, combined with accelerometer-based activity monitoring and with ECG/heart-rate monitoring devices	Standard care without app-based coaching (as specified in the trial design)	Planned 12-week intervention period	Study design: primary outcomes include changes in the daily step count and physical activity; secondary outcomes include functional exercise capacity, cardiorespiratory fitness, biomarkers, and quality of life	Protocol paper – no clinical outcome data reported yet; the trial is designed to test whether MyoMobile-based coaching can increase physical activity and improve functional and biomarker profiles in patients with HFpEF.
Harzand et al., 2023 (PMID: 37700245)	United States	258 patients (from 1,643 referred to CR) with coronary artery disease or heart failure, including patients after myocardial infarction, PCI, CABG and with stable angina,	Phase II–III; home-based remote CR offered as one of several CR modalities	Movn smartphone application combined with a wearable activity tracker and multi-component remote coaching, forming a 3-month patient-centred digital health	Other available CR modalities or usual care among patients referred to CR who did not enrol in the Movn-supported digital intervention	3 months	The 6MWD, LDL cholesterol, smoking status, patient-reported outcomes, and programme completion rate	Among patients enrolled in the Movn-supported digital intervention, 90% completed the programme, with significant improvements in the 6MWD and in LDL cholesterol and a

		referred to CR at a Veterans Affairs medical centre		intervention				reduction in the proportion of current smokers, and no reported adverse events.
Hayn et al., 2023 (PMID: 37519897)	Austria	157 CR patients (107 without supportive tools, 50 using digital tools) after completion of phase III outpatient CR that included a 3–6-month home-training phase; mixed cardiovascular diagnoses typical for outpatient CR	Phase III outpatient CR with a 3–6-month home-based training phase in five outpatient CR centres.	Supportive digital tools used during home training, including digital training diaries and wearables (smartwatches, activity trackers, heart-rate monitoring devices), applied in various combinations.	Patients undergoing the same phase III outpatient CR programme without any supportive digital tools.	Duration of phase III outpatient CR, including 3–6 months of home-based training; retrospective analysis at the end of the phase.	Primary: change in maximum exercise capacity (ΔPmax) during ergometry from pre- to post-phase III; secondary: subgroup differences in ΔPmax according to the type of supportive tool, baseline exercise capacity and risk profile	Both groups improved maximum exercise capacity during phase III CR, with patients using supportive digital tools showing a larger unadjusted gain in Pmax than those without tools (ΔPmax ≈19 vs 9 W), but after adjustment for baseline differences this difference was no longer statistically significant, while subgroup analyses suggested that digital training diaries combined with heart-rate monitoring or activity trackers may confer additional benefits in selected patients
Giggins et al., 2024 (PMID: 39119020)	Ireland	17 (14 men, 3 women; mean age ≈70 years) with a range of cardiovascular disease histories eligible for phase IV (community-based) CR exercise.	Phase IV community-based CR; 8-week exercise programme delivered either online at home or in person at a sports centre.	ECME-CR digital health platform for online CR exercise: web-based app integrated with consumer devices (Withings ScanWatch and BPM Connect) to monitor heart rate, blood pressure and physical activity,	Traditional in-person group CR exercise classes with the same 8-week programme content.	8 weeks (baseline and post-intervention assessments)	Primary: the 6MWD. Secondary: SF-12 physical and mental component scores (health-related quality of life) and qualitative reports of perceived benefits.	Only five participants achieved a clinically meaningful improvement in the 6MWD after the 8-week programme, and no statistically significant changes were observed in objective outcome measures, whereas qualitative interviews

				linked to a backend system (CABIE/SIMS) and used together with live exercise classes delivered via videoconferencing				indicated that participants in both online and in-person groups felt fitter, stronger and reported a range of perceived benefits from taking part in the exercise programme
Wu et al., 2024 (PMID: 39822515)	China	101 patients with first-onset acute myocardial infarction successfully treated with percutaneous coronary intervention, aged 18–70 years, NYHA class ≤ III, able to use smartphone applications.	Early post-discharge CR and continuous care over 6 months; home-based with scheduled outpatient follow-ups	“Internet Plus” continuous remote management model: WeChat-based platform for individualized exercise prescriptions, education (disease, diet, lifestyle, medication), psychological support, weekly online follow-up, medication reminders, and uploading of home BP and exercise logs	Conventional post-PCI care and CR with standard in-person education, exercise prescription, paper exercise logs, and routine follow-up	6 months post-discharge, with assessments at discharge and at 1, 3, and 6 months.	The 6MWD test (exercise tolerance), the Duke Activity Status Index (DASI) score, rehabilitation compliance, anxiety and depression scores (SAS, SDS), incidence of major cardiovascular events, and unplanned rehospitalizations.	Compared with conventional care, the Internet Plus remote-management group showed longer 6MWDs and higher DASI scores at follow-up, higher percentage of excellent rehabilitation compliance (91.5% vs 72.3%), lower anxiety and depression scores at 3 months, and significantly lower 6-month rates of major cardiovascular events (11.1% vs 27.7%) and unplanned rehospitalizations (7.4% vs 23.4%).
Adachi et al., 2025 (PMID: 41034016)	Japan	30 patients who had undergone percutaneous coronary intervention for coronary artery disease, clinically stable and eligible for lifestyle modification and	Early post-PCI secondary prevention phase; 12-week, home-based programme delivered remotely, following a 5-day comprehensive	Web-app-based remote lifestyle modification programme including home-based exercise training, dietary monitoring and health assessments via the	Single-arm pilot; no concurrent control group (participants served as their own controls with pre- vs post-intervention comparisons)	12 weeks of intervention after the initial 5-day assessment.	Systolic blood pressure, lipid profile (LDL-C, HDL-C, non-HDL-C), HbA1c, peak VO_2, moderate-to-vigorous physical activity, the SF-36 mental component summary score, body	The web-app-based programme was feasible, with 80% completion and with no intervention-related adverse events, and was associated with significant reductions in systolic blood

		secondary prevention	lifestyle assessment.	app, with instructors supervising progress, providing feedback and setting goals via an asynchronous chat-based platform.			weight/BMI, and programme completion	pressure, LDL-C, non-HDL-C and HbA1c, significant increases in peak VO_2, moderate-to-vigorous physical activity and SF-36 mental component scores, and meaningful weight loss among participants with elevated baseline BMI.
Oanesa et al., 2025 (PMID: 40847504)	United States	21 participants (mostly CR patients with various eligible cardiac diagnoses and a small number of caregivers) recruited from four academic medical centres; participants with prior or potential indication for CR	Design work for a telehealth CR model intended to complement or substitute centre-based phase II–III CR across multiple sites	Human-centred design of a patient-centred telehealth CR programme and implementation toolkit, including safety protocols, home exercise plans, technology-training scripts, and materials for individual and group telehealth visits (video, phone, messaging, apps)	Not applicable (qualitative/mixed-methods design study; no clinical comparator group).	Not applicable in terms of clinical outcomes; multiple design sessions conducted between January and September 2023	Perceptions of telehealth CR (benefits, barriers, safety, technology usability, social support needs) and co-created programme and implementation materials.	Participants perceived telehealth CR as potentially valuable for personalized home support and convenience but highlighted concerns about technology usability and safety, leading to the co-development of safety protocols, technology-training materials, and an implementation toolkit tailored to diverse patient populations

Table S2. Virtual reality and exergaming systems - detailed study characteristics and outcomes

Author, year, (PMID)	Country	Population and index event	CR phase and setting	Digital intervention	Comparator	Follow-up duration	Main outcomes	Key quantitative results
Blasco-Peris et al., 2025 (PMID: 40669044)	Spain	Small sample of cardiac patients enrolled in outpatient CR (coronary artery disease/after intervention), recruited to test the exergaming prototype during CR sessions	Phase II–III, supervised outpatient centre-based CR with in-clinic testing of the exergaming system.	A prototype exergaming system integrating wearable motion and heart-rate sensors with a game-based interface to guide and monitor exercise intensity during CR sessions.	No formal control group; prototype evaluation alongside standard CR procedures (participants served as their own controls for usability and physiological response)	Short-term feasibility and usability assessment over several exergaming sessions during the CR programme	Feasibility and usability of the exergaming system (task completion, user ratings), safety and adequacy of heart-rate responses during exergaming sessions	The exergaming prototype was successfully integrated into CR sessions, was rated as usable and acceptable by participants, and elicited appropriate exercise-intensity heart-rate responses without intervention-related adverse events, supporting its feasibility for further clinical evaluation
Jóźwik et al., 2021 (PMID: 34440974)	Poland	Patients with coronary artery disease referred to phase II outpatient CR after a recent acute coronary event and/or coronary revascularization; sample size 43 (17:26)	Phase II, supervised outpatient centre-based CR	Virtual-reality–enhanced aerobic exercise sessions with a relaxation component, delivered during centre-based CR using a non-immersive or semi-immersive VR system to provide engaging visual environments during walking or cycling	Standard centre-based CR exercise sessions without VR (usual CR programme)	Duration of the outpatient CR programme (8–12 weeks), with pre- and post-intervention assessments	Functional capacity (the 6MWD test and/or exercise test parameters), patient-reported anxiety and psychological well-being, and safety/tolerability.	Both VR-enhanced and standard CR groups showed significant improvements in exercise capacity over the programme, with the VR group demonstrating at least comparable gains and greater reductions in cardiac rehabilitation–related anxiety and emotional tension,

								without an increase in adverse events
Jóźwik et al., 2021 (PMID: 34065625)	Poland	Adults with coronary artery disease enrolled in phase II outpatient CR after recent acute coronary syndrome and/or coronary revascularization; sample size 100 (50:50)	Phase II, supervised centre-based CR with exercise training on site	Non-immersive or semi-immersive VR system providing interactive virtual environments during cycling/walking exercise sessions to enhance engagement and perceived enjoyment	Standard centre-based CR exercise training without VR (the usual programme)	Duration of the outpatient CR course (e.g. 8–12 weeks), with pre- and post-programme assessments.	Functional capacity (the 6MWD and/or ergometer exercise parameters), perceived exertion and enjoyment, and safety/tolerability; in some analyses, anxiety or quality-of-life measures	Both VR-supported and standard CR groups achieved significant improvements in exercise capacity across the programme, with the VR group showing at least comparable functional gains and higher reported enjoyment of exercise, without an larger-than-average number of adverse events
Szczepańska-Gieracha et al., 2021 (PMID: 33577375)	Poland	Adults with coronary artery disease referred to phase II outpatient CR after an acute coronary event and/or coronary revascularization; sample size 34 (17:17)	Phase II, supervised centre-based CR with structured aerobic exercise sessions.	Virtual-reality–supported exercise sessions using a screen- or headset-based VR system to provide interactive environments during aerobic training (eg, treadmill or cycle ergometer), aiming to increase enjoyment and engagement.	Standard centre-based CR exercise training performed without VR (the usual CR protocol).	Duration of the outpatient CR programme (e.g. 8–12 weeks), with pre- and post-programme assessments	Functional capacity (the 6MWD and/or peak exercise parameters), patient-reported enjoyment, safety and acceptability of exercise.	VR-supported CR produced significant improvements in exercise capacity comparable to standard CR, with higher reported enjoyment of training and no increase in adverse events, suggesting that VR can safely enhance the experiential aspect of outpatient CR without compromising functional benefits
Sibilitz et al., 2022 (PMID:	Denmark, Switzerland,	Patients with cardiovascular	Phase II, supervised	Virtual reality–based relaxation sessions	Standard CR without VR relaxation (the	Duration of the CR programme	Primary: changes in anxiety and	Compared with standard CR alone,

35811477)	the United Kingdom	disease enrolled in a phase II outpatient CR programme after recent cardiac events/procedures; sample size 147	centre-based CR, with additional relaxation sessions	(immersive/semi-immersive calming environments) integrated into the CR schedule to reduce anxiety and improve psychological well-being	usual rehabilitation programme, with or without conventional non-VR relaxation/education)	(several weeks), with baseline and post-programme psychological and functional assessments	other psychological measures. Secondary: functional capacity (e.g., the 6MWD or exercise test parameters) and safety/tolerability	the VR relaxation group showed significantly greater reductions in anxiety scores while achieving similar improvements in functional capacity, with no increase in adverse events, indicating that VR relaxation can safely enhance the psychological benefits of outpatient CR

Table S3. Virtual education platforms and other digital technologies supporting cardiac rehabilitation - detailed study characteristics and outcomes

Author, year, (PMID)	Country	Population and index event	CR phase and setting	Digital intervention	Comparator	Follow-up duration	Main outcomes	Key quantitative results
Brewer et al., 2018 (PMID: 29942596)	United States	8 adults with coronary heart disease eligible for CR, including patients after myocardial infarction and/or coronary revascularization, referred to a CR programme with an educational component	Phase II–III CR; comparison of centre-based CR with in-person education versus CR with web-based/online education	Structured web-based educational platform delivering knowledge about the disease, risk-factors and lifestyle to CR participants as an alternative to conventional face-to-face group education	Standard in-person, centre-based group education sessions as part of CR	Duration of the CR programme (several weeks), with assessments before and after the completion of the educational component.	Disease-specific knowledge, self-reported health behaviours (medication adherence, diet, physical activity), selected cardiovascular risk factors, and, where available, functional measures such as the 6MWD.	Web-based education produced improvements in cardiovascular knowledge and self-reported health behaviours that were comparable to those

								observed with traditional in-person group education, without consistent differences in short-term functional outcomes, which supports online education as a viable alternative format within CR programmes
Lăcraru et al, 2023 (PMID: 36900948)	Romania	50 adults: 30 chronic HF (NYHA II–III) and 20 IHD after coronary events/revascularization with multiple CV risk factors	Post-acute/chronic CR over 3 months: home-based telerehabilitation vs centre-based ambulatory CR vs usual care (HF) and home-based vCare vs usual care (IHD)	vCare virtual assistant (tablet, TV/set-top box, motion camera, wristband, scale, BP monitor) delivering tailored exercise, e-learning, risk-factor and medication-support modules, remotely configured via KIOLA	HF: ambulatory CR and usual-care advice; IHD: usual-care discharge advice without structured CR	3 months with baseline and post-intervention clinical, CPET, lipid, BP, ECG, QoL, psychological and smoking assessments plus usability/acceptance scales in vCare groups.	VO_2max, LDL, QoL (EQ-5D, EQ-VAS, Minnesota), HADS, smoking behaviour, activity metrics and usability/acceptance (UEQ, SUS, TAM)	vCare yielded VO_2max gains comparable to ambulatory CR (HF ~+2 mL/kg/min; IHD ~+4 mL/kg/min), and greater LDL and QoL improvements than usual care; reductions in depressive symptoms and smoking, and usability scores, which indicates acceptable–good user experience and technology acceptance
Indraratna et al, 2022 (PMID: 35225819)	Australia (two tertiary hospitals in Sydney, New South Wales)	164 inpatients recently hospitalized with acute coronary syndrome (78%) or heart failure (22%), randomized at discharge	Early post-discharge / transition from hospital to community over 6 months: smartphone-supported telemonitoring plus	TeleClinical Care (TCC) smartphone app with Bluetooth BP monitor, weight scale and activity tracker, automatic daily data	Usual post-discharge care (GP and cardiologist follow-up; ACS patients	6-month follow-up for readmissions and clinical outcomes, with baseline and 6-month	Primary: 30-day unplanned readmissions; secondary: 6-month all-cause and cardiac readmissions, CR	At 6 months, TCC halved all-cause unplanned readmissions (21 vs 41; HR 0.51) and reduced

			usual care vs usual care alone	upload to the KIOLA platform, the alerting of a cardiologist/nurse team, and thrice-weekly educational push notifications.	referred to standard centre-based CR, HF to outreach services)	assessments including the 6MWD test, LDL, QoL, medication adherence and patient activation	attendance/completion, medication adherence (MGL), QoL (EQ-5D, VAS), Patient Activation Measure, usability rating	cardiac readmissions (11 vs 25; HR 0.44), increased CR completion (39% vs 18%) and good medication adherence (75% vs 50%), with high usability (mean 4.5/5), while 30-day readmissions were similar between groups
Monturo et al, 2025 (PMID: 41342289)	USA (a suburban community hospital within a large regional health system in Pennsylvania)	155 phase II CR graduates (mean age ~68 years; predominantly male, white, non-Hispanic) after mixed cardiac events including PCI with DES, CABG, valve surgery, MI and HF	Centre-based outpatient phase II CR in a multidisciplinary medically supervised cardiopulmonary program, with outcomes reassessed 6 months after program completion (transition into self-regulated phase III)	No structured digital intervention; the study highlights the need to introduce digital technology and eHealth (wearables, home BP/weight monitoring, eHealth tools) during CR II to support long-term maintenance in phase III.	Within-subject longitudinal comparison across three time points (pre-CR, post-CR, 6-month follow-up); no concurrent control group	Three assessments (admission, discharge, 6-month follow-up) including clinical measures, 6MWT, BP, BMI/waist, depression, QoL, diet, smoking, physical activity and medication adherence, plus an online qualitative survey	6MWD, BP, BMI, weight, waist circumference, depression (PHQ-9), QoL (Dartmouth), diet (Rate Your Plate), physical activity (MET-min/week), smoking status and medication adherence.	At 6 months, patients maintained or improved most behavioural and QoL outcomes (physical activity, diet, smoking status, QoL, the 6MWD test, waist circumference) but showed significant rebound in SBP/DBP, weight, BMI, depression scores and slight decline in medication adherence, which prompts a call for integrated digital/eHealth strategies and structured weight-loss support during

								CR II
Wu et al, 2024 (PMID: 39822515)	China (a single tertiary centre, Sir Run Run Shaw Hospital, Zhejiang University School of Medicine, Hangzhou)	101 patients with first-onset acute myocardial infarction treated with successful PCI (NYHA ≤ III, age 18–70), retrospectively assigned to standard care vs "Internet Plus" remote management	Early post-AMI secondary prevention over 6 months: hospital-initiated phase II CR transitioning to home-based phase II/III with continuous follow-up	The "Internet Plus" remote management model using a structured WeChat platform (public account and groups) for individualized exercise prescriptions, diet plans, psychological support, medication reminders, weekly remote follow-ups, digital "punch card" for exercise/BP/medication, and team-supervised data tracking	Routine post-PCI nursing and CR: in-hospital guidance, printed rehab manual, one follow-up call, CPET-based exercise prescription at 1 month, self-logged home exercise and standard outpatient follow-up over 6 months	Assessments at discharge and at 1, 3 and 6 months including the 6MWT, DASI, exercise-compliance classification, SAS/SDS (at discharge and 3 months), and 6-month major cardiovascular events and unplanned rehospitalizations	The 6MWD, DASI functional status, exercise-rehab compliance, anxiety (SAS), depression (SDS), major cardiovascular events and unplanned rehospitalization rates	Compared with standard care, the "Internet Plus" remote management model yielded higher 6MWDs at 1/3/6 months, higher DASI scores at 3/6 months, more patients with excellent CR compliance (≈91% vs 72%), lower anxiety and depression scores at 3 months, and markedly lower 6-month major cardiovascular events (11% vs 28%) and unplanned rehospitalizations (7% vs 23%)
